# Supplementary material for: Leveraging Transformer Models to Capture Multi-Scale Dynamics in Biomolecules by Nano-GPT
Source: J Chem Theory Comput. 2025 Sep 17;21(19):9239–48. doi: 10.1021/acs.jctc.5c00180 (PMC12529894; doi:10.1021/acs.jctc.5c00180)
Supplement: Supplementary file 1 [file ct5c00180_si_001.pdf]

Supporting Information: Leveraging Transformer Models to Capture Multi-Scale Dynamics in Biomolecules by nano-GPT

Wenqi ZENG,[†] Lu ZHANG,[‡] and Yuan YAO[*,†,¶]

†*Department of Mathematics, The Hong Kong University of Science and Technology, Clear Water Bay, Kowloon, Hong Kong SAR 999077, China*

‡*State Key Laboratory of Structural Chemistry, Fujian Institute of Research on the Structure of Matter, Chinese Academy of Sciences, Fuzhou, Fujian 350002, China*

¶*Department of Chemical and Biological Engineering, The Hong Kong University of Science and Technology, Clear Water Bay, Kowloon, Hong Kong SAR 999077, China*

E-mail: yuany@ust.hk

1 Detailed Structure of Transformer Layer

A pivotal component of the GPT architecture is the self-attention mechanism. This mechanism transforms the input embeddings $\mathbf{X}$ into hidden vectors $\mathbf{H}^{(l)}$ through following steps:

Given query matrix $\mathbf{W}_Q \in \mathbb{R}^{D_q \times D_x}$, key matrix $\mathbf{W}_K \in \mathbb{R}^{D_q \times D_x}$ and value matrix $\mathbf{W}_V \in \mathbb{R}^{D_v \times D_x}$, the $\mathbf{X}$ is projected into $\mathbf{Q}, \mathbf{K}, \mathbf{V}$ as:

$$\mathbf{Q}^{(l)} = \mathbf{H}^{(l-1)}\mathbf{W}_Q^T,$$
$$\mathbf{K}^{(l)} = \mathbf{H}^{(l-1)}\mathbf{W}_K^T,$$
$$\mathbf{V}^{(l)} = \mathbf{H}^{(l-1)}\mathbf{W}_V^T$$

where $\mathbf{H}^{(0)} = \mathbf{X}$ and we denote $\mathbf{Q} := [\mathbf{q}_1, .., \mathbf{q}_t]^T$, $\mathbf{K} := [\mathbf{k}_1, .., \mathbf{k}_t]^T$, $\mathbf{V} := [\mathbf{v}_1, .., \mathbf{v}_t]^T$ for $i = 1, ..., t$.

The output for next layer $\mathbf{H}^{(l)}$ is then defined as the same equations in the main text:

$$\mathbf{H}^{(l)} = \mathbf{H}^{(l-1)} + \mathbf{A}^{(l)} + \mathbf{B}^{(l)}, \tag{1}$$
$$\mathbf{A}^{(l)} = softmax(\frac{\mathbf{Q}^{(l)}(\mathbf{K}^{(l)})^T}{\sqrt{D_q}})\mathbf{V}^{(l)}, \tag{2}$$
$$\mathbf{B}^{(l)} = f_\theta(\mathbf{H}^{(l-1)} + \mathbf{A}^{(l)}) \tag{3}$$

From the left to right in Eq. 3, the addition of $\mathbf{H}^{(l-1)}$ stands for the residual connection inspired by ResNet. In Eq. 3, $\mathbf{A}$ is also known as attention matrix, which stands for the contextual information learned for every token in input sequence. The softmax function is applied row-wise on $\frac{\mathbf{QK}^T}{\sqrt{D_q}}$. For each vector in $\mathbf{A}$, an equivalent form is given as $\mathbf{A} := [\mathbf{a}_1, ..., \mathbf{a}_t]$ where:

$$\mathbf{a}_i = \sum_{j=1}^{t} softmax(\mathbf{q}_i^T\mathbf{k}_j/\sqrt{D_q})\mathbf{v}_j$$

The $\mathbf{B}^{(l)}$ in Eq. 3 stands for the MLP network in the structure for nano-GPT, which is consisted of a two-layer neural network and normalizing non-linear networks. For the simplicity of notation, we use f_θ to characterize the non-linear transformation. Recall $\mathbf{H}^{(0)}$ is initialized as $\mathbf{X}$, $\mathbf{H}^{(l)}$ can be rewritten as:

$$\mathbf{H}^{(l)} = \mathbf{X} + \sum_{k=0}^{l}(\mathbf{A}^{(k)} + \mathbf{B}^{(k)}) \tag{4}$$

The decoding steps that yield the final probability distribution $\mathbb{P}(\hat{x}_{t+1}|x_1, ..., x_t)$ involve a feed-forward neural network, which incorporates layer normalization and a residual connection. To simplify the notation, we represent this process as a nonlinear function f_θ. Following f_θ, the model employs a linear projection, represented by $\mathbf{D} \in \mathbb{R}^{D_{out} \times D_x}$, and concludes with a softmax activation function. This sequence of operations effectively transforms the hidden representations into a probability distribution over potential output tokens.

2 Experimental Details

2.1 Hyperparameters

The exponential-decay scheme has been studied in[S1] with code in `https://github.com/Adaxry/ss_on_decoding_steps`. The scheduled sampling strategy used in our work follows the "composite setting" described in Section 3.4 of the paper, which combines two exponential functions : one for the training epoch i and another for the decoding step t .

1. Decay Function for Training Epoch i: We employ an exponential decay scheme: $f(i) = k^i$. This approach was found to be the top performer in Table 5 from[S1] when compared among linear, exponential, and sigmoid decay schemes.

2. Decay Function for Decoding Step t: Similarly, for the decoding step, we use: $g(t) = \epsilon^t$ which follows an analogous exponential decay pattern.

3. Combination of $f(i)$ and $g(t)$: We have choices among *Product* $h(i,t) = f(i) \times g(t)$, *Mean* $h(i,t) = \frac{f(i)+g(t)}{2}$, *Composite* $h(i,t) = g(t(1 - f(i)))$. The key insight behind this choice is that it maintains a progressive balance between using golden tokens and model-generated predictions as shown in Fig. S1. When in early steps of training or decoding (yellow circle in Fig. S1), more ground truth samples are used in training to provide strong supervision. Later in training and at deeper decoding steps, the model

relies more on its own outputs, improving robustness during inference. The *composite* setting provide the desired probability pattern, compared to *product* and *mean* setting.

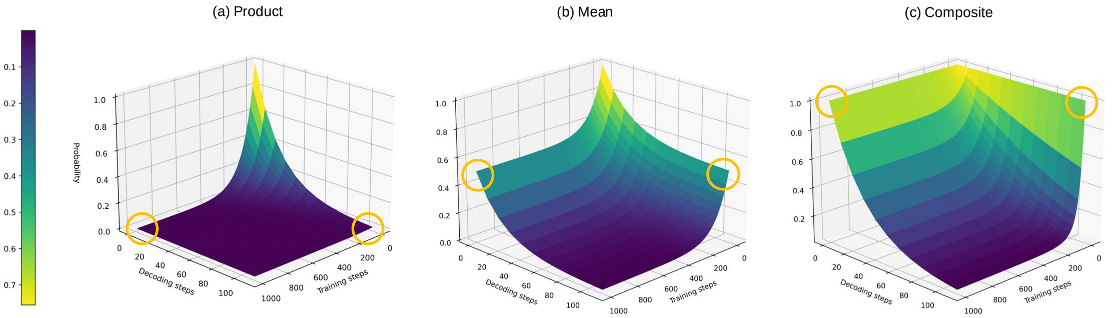

Figure S1: Examples for different form of h(i,t): product, mean and composite. The closer the color to yellow, the greater the probability of sampling golden tokens. Orange circles are for the sake of highlights.

The parameters ϵ and k are treated as hyperparameters and selected based on experimental validation. The specific hyperparameters used in this paper are listed in Table. S1:

The experiments for nano-GPT and LSTM are conducted under fair and comparable settings. To ensure this, both models were trained until convergence, as monitored by the validation loss. The models were trained for a comparable number of epochs, and their hyperparameters (shown in Table S1) were tuned via random search. After selecting the best-trained model based on validation performance by loss and accuracy metric, we saved the checkpoints for the top 3 epochs and used them to run independent simulations under fixed random seeds. Both nano-GPT and LSTM generated 20 trajectories of length 100,000. We also verified that neither model suffered from underfitting by comparing training and validation curves.

2.2 Datasets

Table S2 summarizes the datasets employed in our experiments, detailing the number of trajectories, datapoints per trajectory, and state definitions. All datasets are split into training, validation, and test sets with a ratio of 0.7/0.1/0.2. The MFPT (Mean First

Table S1: Hyperparameters used for each dataset across different models. Shared parameters are listed separately from model-specific configurations for nano-GPT and LSTM.

**Model**	**Parameter**	**4-state**	alanine$_{\psi\&\phi}$	alanine$_{RMSD}$	**Fip35 WW**
Shared	Number of States	4	20	100	100
	Training Window Size	100			
	Train / Val / Test Split	0.7 / 0.1 / 0.2			
nano-GPT	Learning Rate	0.0001	0.0005	0.001	0.0005
	Number of Blocks	1	2	2	3
	Hidden Dimension	64	128	64	128
	k (Scheduled Sampler)	0.9999	0.9995	0.9995	0.9995
	ϵ (Scheduled Sampler)	0.995	0.95	0.95	0.95
	Batch Size	32	32	32	32
	Shared Parameters	TRUE	TRUE	TRUE	TRUE
LSTM	Learning Rate	0.001	0.001	0.001	0.0005
	Hidden Dimension	8	128	128	64
	Units	64	512	512	256
	Stateful	TRUE	TRUE	TRUE	TRUE
	Batch Size	64	64	32	32

Passage Time) values between selected state pairs are reported for each dataset. For the alanine dipeptide systems, states A and B correspond to α_L and C_5, respectively, while in the Fip35 WW domain, state A represents the "random coil" state and state B represents the "folded" state, as illustrated in Fig. 5 of the main text.

Table S2: Summary of datasets used in our experiments. All datasets are split into training, validation, and test sets with a ratio of 0.7/0.1/0.2. MFPTs (Mean First Passage Times) are reported between selected state pairs. For alanine dipeptide systems, state A corresponds to α_L and state B to C_5. For the Fip35 WW domain, state A denotes the "random coil" state and state B denotes the "folded" state, as illustrated in Fig. 5 of the main text.

**Dataset**	**# Traj.**	**Datapoints / Traj.**	**Interval**	**# States**	**MFPT$_{A\to B}$**	**MFPT$_{B\to A}$**
4-state	1	1,600,000	–	4	–	–
alanine$_\psi$	100	100,000	0.1 ps	20	153 ps	89 ps
alanine$_\phi$	100	100,000	0.1 ps	20	984 ps	30,836 ps
alanine$_{\mathrm{RMSD}}$	100	100,000	0.1 ps	100	5,417 ps	84,830 ps
Fip35 WW	57	100,000	0.2 ns	100	18 ns	5 ns

3 Ablation Study

Scheduled Sampler: In this part, we show the ablation study on the scheduled sampler in $\textbf{alanine}_{\textbf{RMSD}}$, to prove that the sampler block is important for capturing accurate dynamics. The result shows that, the adding of scheduled sampler is essential for predicting MFPT and ITS.

Both the scheduled sampler and Nano-GPT's attention mechanism contribute to modeling long-range dependencies, but they serve different roles. Scheduled sampling gradually shifts from using ground-truth tokens to model predictions during training, mitigating exposure bias[S2] and improving robustness during sequence generation. This technique benefits both LSTM and Transformer-based models by better aligning training and inference conditions[S3]. In contrast, nano-GPT's attention mechanism enables direct access to all previous time steps, allowing the model to learn dependencies over arbitrarily long ranges more effectively than sequential models like LSTM.

Previous studies[S4] showed that scheduled sampling improves lexical accuracy in LSTM-based models, and Zhou et al.[S3] demonstrated that LSTM with scheduled sampling outperforms vanilla LSTM in automatic speech recognition tasks (e.g., in Table 2, configuration E1 achieves a lower Character Error Rate (CER) than B1). However, the best results are achieved when both scheduled sampling and transformer-based architectures are combined (see Table 3, configuration E8+E3), highlighting the unique advantage of attention mechanisms in modeling complex temporal dependencies.

To address this more directly, we have added an ablation study (Table S3) comparing LSTM and Nano-GPT models trained with scheduled sampling. LSTM with scheduled sampling outperforms vanilla LSTM, especially on the transition from faster dynamics α_L to β. However, the best results are achieved when both scheduled sampling and GPT-based architectures are combined, highlighting the unique advantage of attention mechanisms in modeling complex temporal dependencies. Attaching a scheduled sampler to LSTM alone

is unlikely to match the performance of nano-GPT in tasks requiring managing long-range dependencies.

Table S3: Ablation study of scheduled sampling (ScheSamp) on the alanine dipeptide system, comparison between nano-GPT and LSTM. MFPTs (Mean First Passage Times) from β to α_L and from α_L to β are reported, along with their standard deviations.

**Model**	**Setting**	$\beta \to \alpha_L$	std	$\alpha_L \to \beta$	std
MD	Ground Truth	127,836	–	1,502	–
nano-GPT	w/ ScheSamp	110,916	± 23,045	1,568	± 193
	w/o ScheSamp	59,367	± 6,738	906	± 72
LSTM	w/ ScheSamp	96,524	± 20,352	985	± 104
	w/o ScheSamp	48,438	± 23,129	655	± 64

Number of States: Regarding model performance, Table. S4 shows that increasing the number of discrete states leads to consistently robust predictive accuracy. While finer discretization adds resolution, the implied timescales remain stable and accurately captured across all tested settings: 5, 100, and 300 states. The 5-state setting can be interpreted as a coarse-grained macrostate model, where it is important to ensure a clear separation of timescales. In contrast, using 100 or 300 microstates treats the number of clusters as a tunable hyperparameter. For the experiments presented in the main text, we selected 100 states as a balanced choice. The 100-state setting avoids the ambiguity of defining separable macrostates in the 5-state case, while also limits computational overhead compared to the 300-state setting.

Table S4: Ablation study of different number of states for Fip35 WW domain. The ITS are calculated with a lagtime of 20 ns, where the lag time corresponds to the discrete time interval between observations in the trajectory used for computing transition probabilities or identifying state transitions. The ± error stands for the standard deviation computed over 3 independent simulations for nano-GPT.

Dataset / Model	Number of States	1st ITS	2nd ITS	3rd ITS
Ground Truth MD	5	15,228	1,962	249
	5	12,169 ± 2,090	2,674 ± 258	319 ± 37
nano-GPT	100	16,853 ± 1,972	2,549 ± 241	501 ± 60
	300	14,874 ± 1,176	1,224 ± 201	188 ± 24

Window Size: Table S5 shows that increasing the training window size significantly improves the performance of both nano-GPT and LSTM, particularly for LSTM. When the window size is small (100 or 200), LSTM performs poorly, with large deviations from MD ground truth and high variance. In contrast, nano-GPT remains relatively stable and closer to ground truth across all settings. With a longer training window (1000), LSTM shows improved performance and reduced variance, indicating better modeling of long-range dependencies. However, nano-GPT consistently achieves lower errors and variance overall, showing stronger robustness to different training window sizes."

4 Supplementary Experiments

4.1 Transitions in 4-state system

To further illustrate the energy barrier between the AD and BC pair, Fig. S2 and Fig. S3 analyzes the transition count as a function of commit time. States with significant energy barriers often involve rare events that can be overlooked by the model. Despite their infrequent occurrence, transitions across these barriers are of great interest.

By setting all y-axes to the same range, result shows that transitions between states A and D are rare, whereas transitions such as A to C and B to D occur more frequently.

Table S5: Ablation study of different training window size on $\text{alanine}_{\text{RMSD}}$. For both nano-GPT and LSTM models, the table presents the average values (Mean) and their corresponding standard deviations (Std) derived from five independent runs. MD ground truth has no Std. Values closer to the MD ground truth reflect better performance.

**Train Window**	**Method**	$\boldsymbol{\alpha_l \to \alpha_r}$		$\boldsymbol{\beta \to \alpha_l}$		$\boldsymbol{\alpha_l \to C_5}$		$\boldsymbol{C_5 \to \alpha_l}$	
		Mean	Std	Mean	Std	Mean	Std	Mean	Std
100	nano-GPT	61,983	±9,220	62,013	±9,194	5,951	±52	62,013	±9,185
	LSTM	57,100	±17,232	57,121	±17,239	10,307	±1,326	57,121	±17,242
	MD	84,921	–	84,836	–	5,417	–	84,830	–
200	nano-GPT	110,899	±28,012	110,916	±23,762	4,728	±808	110,919	±23,762
	LSTM	48,422	±23,167	48,438	±23,129	4,968	±44	48,438	±23,132
	MD	127,178	–	127,088	–	9,760	–	127,078	–
1000	nano-GPT	82,653	±5,053	82,615	±5,058	9,430	±737	82,613	±5,058
	LSTM	78,421	±15,961	78,377	±15,939	9,944	±242	78,370	±15,942
	MD	84,903	–	84,888	–	8,722	–	84,882	–

Both nano-GPT and LSTM effectively capture these frequent transitions. However, for rare transitions—such as A to D, as highlighted in the main text with a closer view—nano-GPT more accurately models the near-zero transition counts, while LSTM shows greater deviation from the ground truth.

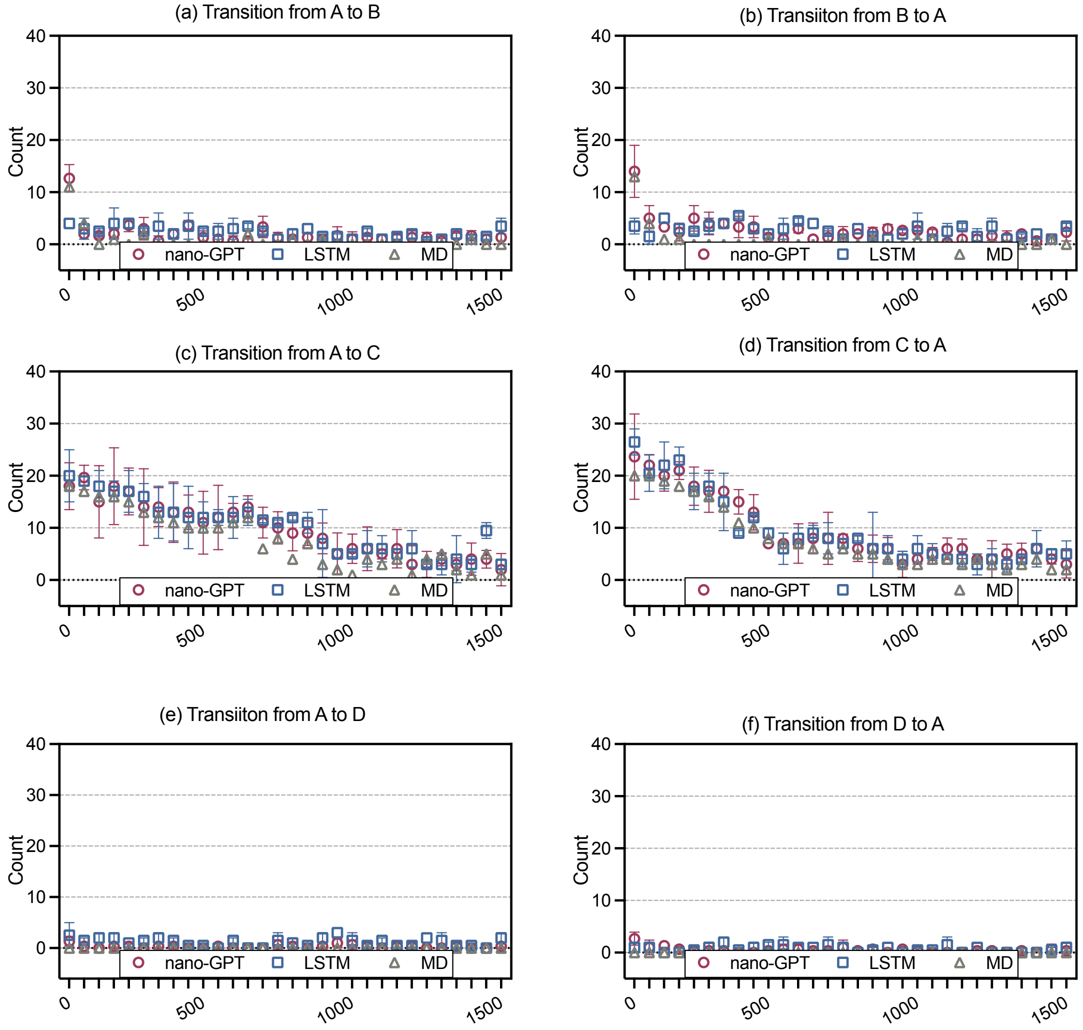

Figure S2: Transition Counts Between 6 State Pairs in the 4-State Model Potential, AB, AC and AD. Error bars are illustrated and calculated as the standard deviation for 3 independent runs.

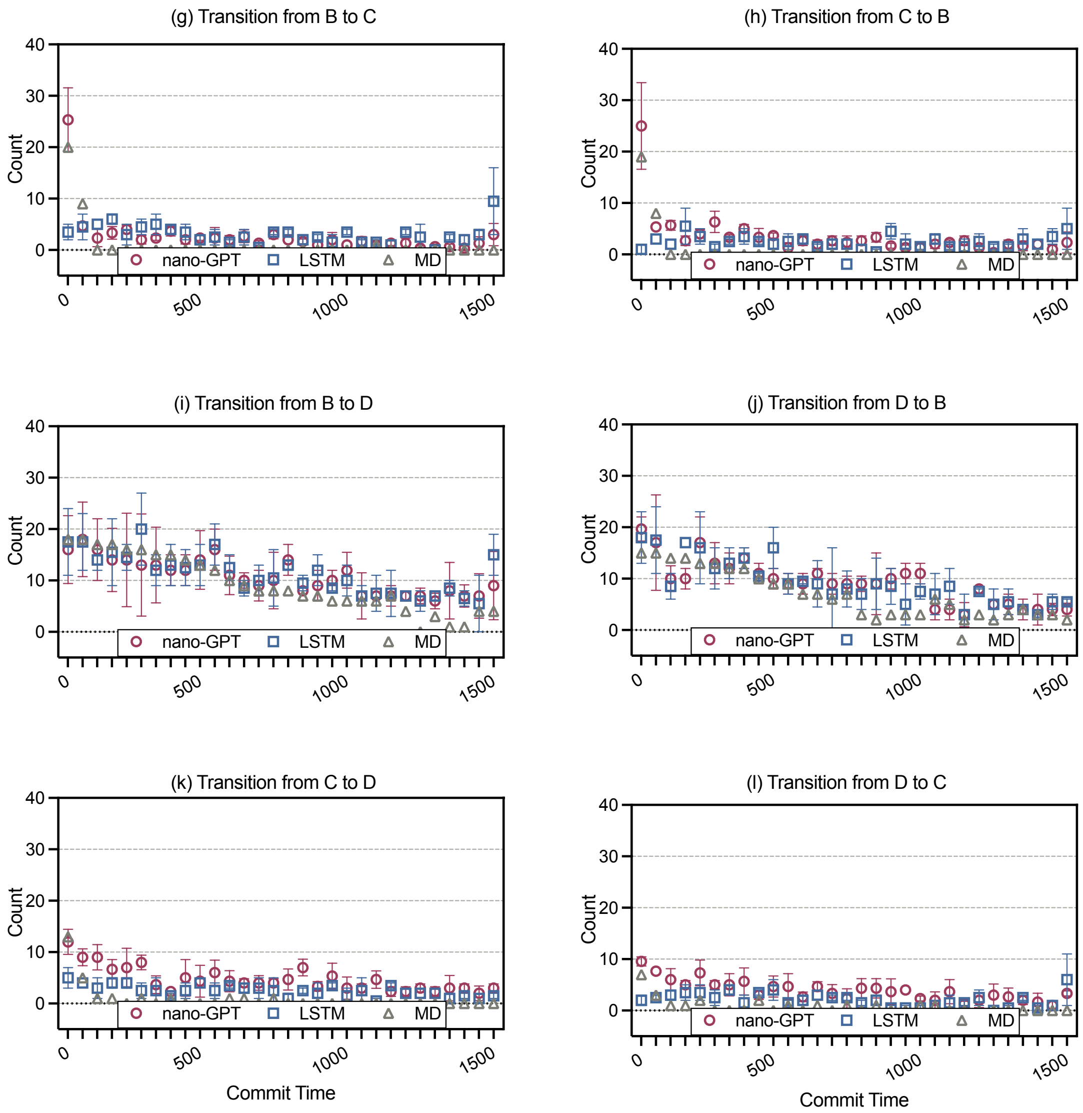

Figure S3: Transition Counts Between 6 State Pairs in the 4-State Model Potential, BC, BD and CD. Error bars are illustrated and calculated as the standard deviation for 3 independent runs.

4.2 Causal Trace in Alanine Dipeptide

We provide an analysis on nano-GPT of its interior mechanism in comprehending long MD trajectories. The challenge of long sequences stems from the dynamics staying in one state for extended duration, where LSTM may encounter memory leakage issues despite the presence of gating mechanisms. To address this enhanced ability of nano-GPT in dealing with long

sequences, we employ causal trace technique[S5] to investigate the information flow in nano-GPT.

(i) Initially, the model is run normally to obtain the predicted result, denoted as $\hat{x}_{t+1}$, with input X processed as depicted in model structure from main text.

(ii) In the next step, the embeddings $\mathbf{H}_i^{(0)}$ are corrupted by adding noise for all index i, specifically $\mathbf{H}_i^{(0)}+\epsilon$, where ϵ follows a normal distribution $N(0,\sigma)$. This results in a corrupted prediction, denoted as $\hat{x}^*_{t+1}$.

(iii) To assess the causal effect, in the third step, at a given token i and layer l, the embeddings $\mathbf{H}_i^{(l)}$ are restored to their clean, noise-free state without ϵ. An important state should have the capability to recover $\hat{x}_{t+1}^{clean\mathbf{H}_i^{(l)}}$ instead of $\hat{x}^*_{t+1}$.

To quantify the causal effect, we define the Total Effect (TE) as $TE = \mathbb{P}(\hat{x}_{t+1}) - \mathbb{P}(\hat{x}^*_{t+1})$. Additionally, we use the concept of Indirect Effect (IE) to measure how $\mathbf{H}_i^{(l)}$ influences the prediction when compared to the corrupted version: $IE = \mathbb{P}(\hat{x}_{t+1}^{clean\mathbf{H}_i^{(l)}}) - \mathbb{P}(\hat{x}^*_{t+1})$. These measures allow us to assess the extent of influence and mediation that different components have on the final prediction.

The results presented in Fig. **??** provide insights into the direct probabilities when restoring embeddings or attention layers on a single sample sequence. In this case, the target prediction is the α_l state, and it's expected that states in positions preceding it will have a significant impact on the final prediction. This is reasonable considering that the attention mechanism considers every position without suffering from memory loss. Interestingly, metastable state 87 (β state) is found to contribute significantly to enhancing the probability for accurate prediction. This observation underscores nano-GPT's ability to capture intrinsic transitions within the entire dynamic.

4.3 High Order Dependency

While MD simulations explore new physical states by sampling conformational space, nano-GPT, as a language model, operates within predefined states, learning and reproducing the

statistical properties of data. While it does not generate physically novel states, the attention mechanism in nano-GPT offers a unique advantage: it captures high-order dependencies and long-range correlations between states, which may be difficult to model using traditional approaches such as MSMs. Our results show that earlier states can influence distant states, providing a complementary perspective to understanding molecular dynamics. While nano-GPT does not autonomously discover new states, it offers valuable insights into complex dependencies across time.

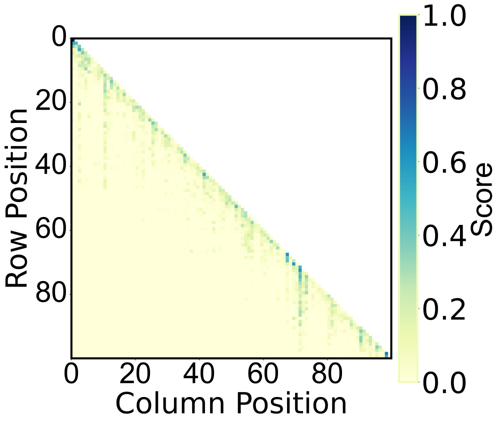

Figure S5: Attention map in nano-GPT for a short segment (length 100) from the Fip35 WW domain trajectory. The shaded regions indicate strong attention weights from the column position to the row position, highlighting high-order dependencies among states within the trajectory.

4.4 Fast Dynamics

Fig. S6 presents a complementary analysis showing how fast dynamics are preserved in the simulation of nano-GPT, but may be missed in MSMs. We consider four settings: *empirical MFPT* for the MD trajectory, *empirical MFPT* for nano-GPT, MFPT calculated from the transition probability matrix (TPM) of the MSM, and *empirical MFPT* from random sampling based on the MSM.

The *empirical MFPT* is computed by counting the first passage times between two states and taking the average. For the nano-GPT simulations and random sampling from the MSM, we perform 3 independent simulations, each generating 20 trajectories. The test system is

alanine dipeptide with 100 microstates, where the fast dynamics refers to transitions from state A to state B, as shown in Fig. S6(a). These states correspond to the left and right regions of the β basin.

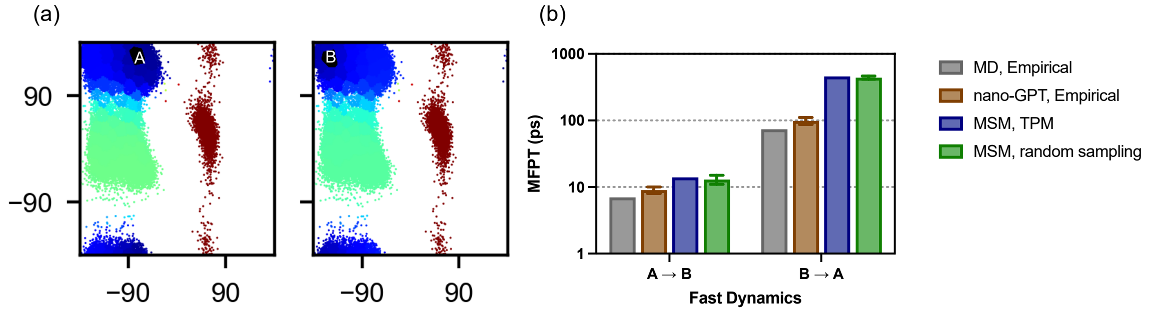

Figure S6: Fast dynamics of alanine dipeptide.(a) Ramachandran plot for states A and B, where A is left region of β, B is right region of β. (b) The MFPT results for fast dynamics from A to B, and B to A, error bars stand for the standard deviation for 3 independent runs.

The results in Fig. S6 show that the fast dynamics produced by MSM random sampling deviates significantly from the empirical results, whereas nano-GPT's simulations closely capture fast dynamics observed empirically. This distinction highlights the novelty of nano-GPT in capturing richer dynamics than MSMs can represent. The phenomenon may be attributed to the fact that MSMs rely on the assumption of metastable states and are designed to capture long-term dynamics by ensuring the system reaches Markovian equilibrium or steady implied time scales. In contrast, our model, nano-GPT, does not require any prior assumption regarding state discretization or Markovian behavior. This distinction allows nano-GPT to learn fast dynamics directly from the data, including intermediate or transient states.

Furthermore, MSMs focus on capturing dynamics based on coarse-grained meta-states, nano-GPT can model the dynamics from microstates, allowing for a more detailed representation of the underlying system. Although in our evaluation, we use metrics that capture meta-dynamics, but the dynamics learned by nano-GPT are inherently different and richer because they consider more detailed interactions, including those from intermediate states.

Therefore, rather than a direct comparison with MSMs, we propose that the unique contribution of nano-GPT lies in its ability to model complex, non-Markovian dynamics, and its flexibility in learning from both metastable and intermediate states without strict predefined assumptions.
